# The effects of China’s New Cooperative Medical Scheme on accessibility and affordability of healthcare services: an empirical research in Liaoning Province

**DOI:** 10.1186/1472-6963-14-388

**Published:** 2014-09-13

**Authors:** Xin Wang, Xin He, Ang Zheng, Xianpu Ji

**Affiliations:** Department of the Health Service Management, China Medical University, No.92 North Second Road, Heping District, Shenyang, Liaoning Province 110001 China (PRC; China Medical University seven-year system, China Medical University, No.92 North Second Road, Heping District, Shenyang, Liaoning Province 110001 China (PRC)

**Keywords:** Case study, New Cooperative Medical Scheme, Designated hospitals, Inadequate and overly expensive medical services

## Abstract

**Background:**

China’s New Cooperative Medical Scheme (NCMS), launched in 2003, was intended to prevent the impoverishment due to catastrophic illness costs. Previous studies have been conducted on the “design flows” of the NCMS, for example, the irrational insurance benefit package. But after several years of implementation, very little has been known about the improvements made by the NCMS and rural residents’ attitudes toward it. This article specifically focused on the improvements of healthcare services and the enrollees’ choices of providers since the implementation of the NCMS in Liaoning province.

**Methods:**

We conducted a one-on-one interview with healthcare officials in order to get a better understanding of the NCMS policies of the local area. We conducted a door-to-door survey in 3 counties, 21 villages and 602 households to gauge population characteristics, respondents’ healthcare preferences, satisfaction levels with providers, and their attitudes towards designated healthcare institutions.

**Results:**

We found that 43.6% of the respondents believed the NCMS brought more convenience to receive healthcare services. 35.2% of the rural residents thought the NCMS work ineffectively, mainly due to the high healthcare costs. 72.3% of the respondents preferred the county hospitals when they got severe diseases, mainly for the reason of better skills and more advanced equipment, while they preferred village clinics (56.5%) and township hospitals (23.2%) when they got minor diseases mainly for the reason of convenience.

**Conclusion:**

We concluded that the NCMS improved the situation of hard to receive healthcare services but did not reduce the high healthcare fees. Furthermore, participants were unsatisfied with the NCMS designated hospitals. Based on our findings, a number of remedial actions were proposed, including redistributing healthcare resources, developing more domestic medical equipment to lower the treatment costs, and establishing a new talent flow mode.

## Background

As one of the largest developing countries in the world, China has been striving to create a universal healthcare system. The healthcare insurance has the potential to prevent the inaccessibility to healthcare services due to financial reasons, since the financial risk of healthcare services is shared among insurance enrollees and the healthcare costs will be reduced as well [1]. Currently, the primary healthcare insurance programs in China could be divided into three categories, namely, the Urban Employee Basic Medical Insurance (UEBMI) for urban employees, the Urban Resident Basic Medical Insurance (URBMI) for urban residents, and the New Cooperative Medical Scheme (NCMS) for rural residents.

According to the 6th national population census in China, by 2010 the total rural population had reached 674 million, accounting for 50.3% of the country’s total population of 1.339 billion. Rural public health has become one of the top priorities in China’s healthcare system, and how to improve rural residents’ health and equalize the public health services has been gaining great attention [2].

Since established in 2003, the NCMS was designed exclusively for the rural population according to their hukou, with the aim of improving accessibility to healthcare services and preventing catastrophic illnesses [3–5]. Before its implementation, by the early 2000s, 65% of the rural residents requiring hospitalization were either opting not to be admitted or checking themselves out of care before their doctors recommended discharge, with the main reason of financial concerns [6]. And according to the third National Health Service Survey in 2003, 80% of the rural residents were not covered by any form of healthcare insurances and 30% of patients could not afford hospitalization when they needed it [7]. With 304 pilot counties, the NCMS is organized, guided, and largely funded by both central and local governments, and only requiring its participants to pay a small amount of money (approximately US$1.50) to the Bureau of Health in their county government [8]. Instead of at the village or township level, NCMS operates at the county level; thus, it provides a larger risk pool and economies of scale in organization and management [9]. The ministry of health of central and local government agencies is the policy maker and mainstay of NCMS. Additionally, the financing mechanism has delegated the management of the NCMS to local governments, and differences in funds raised and reimbursement rates between regions were permitted [10].

The rural healthcare providers could be classified into county hospitals, township hospitals and village clinics, with the county hospitals on the top and the village clinics at the bottom [11]. In addition, for patients requiring senior healthcare services, a transferal policy has been established to help the patients transfer from designated county hospitals to city/provincial hospitals. Generally, as the funding of the NCMS is based on the county level of governmental organization, it is expected that people will seek healthcare services in designated hospitals, most of which are located within the home county; while for enrollees out-of-county, reimbursement for city services is severely limited [4]. Furthermore, healthcare services are provided through a fee-for-service reimbursement with the focus on inpatient care, and the reimbursement rates vary by different types of care and at different health facilities. In 2007, the NCMS has extended its coverage to outpatient care, with the emphasis on catastrophic outpatient costs, and the reimbursement is made through participants’ Medical Saving Account or pooled funds [12]. In addition, the NCMS only reimburses drugs listed on the National Essential Drug Reimbursement List, services covered by the insurance package, and care sought at state-owned public health facilities. The reimbursement rates, though different in each province, are the highest for care delivered at village/township health centers and the lowest at city/provincial hospitals [13, 14].

Initial success has been observed in the aspect of policy promotion. By 2010, more than 96% of the rural residents were covered by the NCMS [15], and about 90% of the participating rural households expressed their willingness to continue to participate the program [16]. After nine years of implementation, according to the 2012 Report on the Work of the Chinese Government, the NCMS has covered 832 million people, that is 97.5% of rural resident. Furthermore, government contribution to insurance premium increased from 10 RMB (US$1.60) in 2003 to 240 RMB (US$38.51) in 2012; and insurance packages have expanded from covering mainly catastrophic illness to outpatient and prevention care [17].

To date, a number of studies have been conducted on the “design flows” of the NCMS. Yip et al. have demonstrated the irrational NCMS packages that ignored the disease profile and health expenditure pattern of the population, which would limit the effectiveness [18]. But little has been known about how far the NCMS can improve access to healthcare services and the overall effect of the NCMS on the beneficiaries. In our study, we focused on the effects of the NCMS on the healthcare accessibility and affordability, the rural residents’ attitudes toward the designated healthcare institutions and their choices of providers.

We interviewed NCMS policy makers in 19 counties in Liaoning province, as well as the executors of related village, township and county hospitals. In addition, we extensively collected opinions from the primary-level staff on their views of the present rural health policies. Later we randomly selected 3 counties with high, middle and low level of economic development, respectively. We conducted a survey with the rural residents in these counties on their opinions of the NCMS, expecting to find the factors that influence the healthcare service quality and the improvements of the NCMS.

## Methods

### Study sample

There are 19 counties in Liaoning province, and we subclassified them into 3 groups according to the average income. Dawa County, Changtu County and Xinbin County were selected through stratified sampling. From each of the selected counties, seven villages were randomly chosen. In each county, 30 households were randomly selected. We conducted a door-to-door survey with the target households. The sample size was calculated as 2 ‰ of the total number of households in each area. The survey was conducted with trained interviewers in 3 counties, 21 villages and 630 households. We got 630 questionnaires in total, and 602 were qualified. Informed consent was obtained from all participants following a protocol approved by Ethics Committee of China Medical University.

There were two parts in our study: interview and survey. One-on-one interview was conducted with the health officials with administrative responsibilities at city, county, township and village levels in order to get a better understanding of the local NCMS policies. A door-to-door survey was undertaken with a locally contextualized questionnaire, containing customized question sets designed to gauge population characteristics, respondents’ healthcare preferences, satisfaction levels with providers, and opinions on designated healthcare institutions in general. All the interviewers were graduate students who have received detailed in-person training.

### Data analysis

Data extraction was checked through parallel double entry with Epidata 3.0. Demographic characteristics of respondents including age, gender, family size, education background, job, annual income, medical expenses, etc. were described (Table 1). Analyses were performed with SPSS 13.0 and Excel.

**Table 1:** **Basic information of surveyed individuals/families**

Variables		N	Percentages
SEX	Male	218	36.2
	Female	384	63.8
AGE(YEARS)	≤40	110	18.3
	40-49	186	30.9
	50-59	190	31.6
	≥60	116	19.3
FAMILY SIZE (NUMBER OF FAMILY MEMBER)	1	37	6.1
	2	124	20.6
	3	195	32.4
	4	131	21.8
	≥5	115	19.1
EDUCATION BACKGROUND	No schooling	68	11.3
	Primary school	113	18.8
	Junior high school	300	49.8
	Senior high school	110	18.3
	College/beyond college	11	1.8
JOB	Professionals	48	8.0
	Farmers	285	47.3
	Unemployed or contract workers	217	36.0
	Workers or businessmen	52	8.6
ANNUAL INCOME	≤3000	42	7.0
	3000-8000	125	20.8
	8000-12000	140	23.3
	12000-20000	158	26.2
	≥20000	137	22.8
MEDICAL EXPENSES	<500	122	20.3
	500-5000	358	59.5
	5000-10000	83	13.8
	>10000	39	6.5
MEDICAL EXPENSES/ANNUAL INCOME	≤20%	360	59.8
	20%-40%	111	18.4
	≥40%	131	21.8

## Results

### General data

In our sample, the two-week prevalence rate^a^ (except chronic diseases) was 12.4%, lower than the national level in 2008 (18.9%); the hospital admission rate was 17.2%; the choices of first clinical hospital were: county hospitals (46.7%), and township hospitals or village clinics (43.3%); 54.8% of the households were diagnosed with chronic diseases, with the cardiovascular diseases being top at 28.9%, followed by diabetes (14.3%), digestive diseases (3.2%), chronic pulmonary diseases (2.4%), lumbar and cervical diseases (2.7%) and mental diseases (1.1%). A considerable number (37.2%) of the surveyed households had at least one family member who had suffered from the diseases requiring high healthcare fees; 5.5% of these families could not afford the high fees and 32.8% could barely make it.

### Inadequate and overly expensive healthcare services

**Figure 1:**
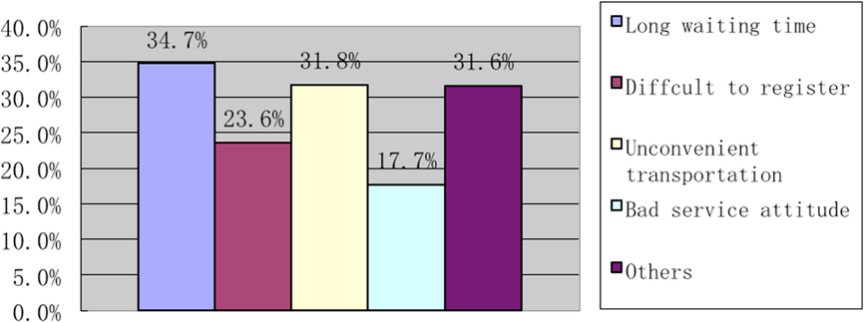
**Main reasons for inadequate healthcare services.**

**Figure 2:**
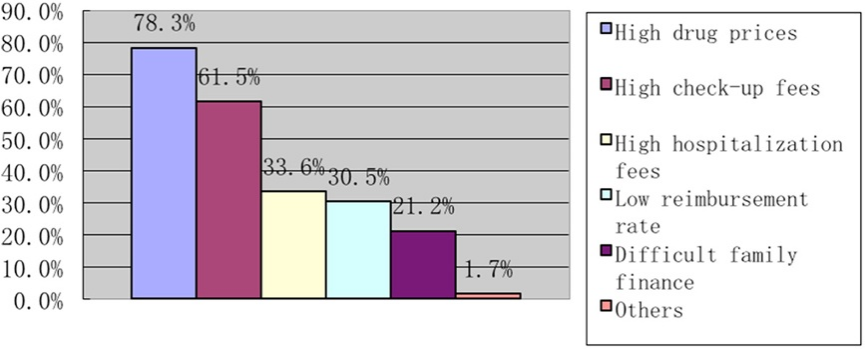
**Reasons for high healthcare services fees.**

According to our study, 24.6% of the participants held that the long waiting time had been the major concern in healthcare services (Figure 1). Other reasons (31.6%) accounting for inadequate healthcare services were: the regular work-and-rest time of designated healthcare institutions is inconvenient for farmers to see a doctor; it is difficult to transfer to another hospital; the medical staff and drugs are inadequate at designated hospitals. In addition, 32.7% of the participants complained of poor accessibility to city hospitals; 25.3% of the participants had poor accessibility to county hospitals; and17.9% of the participants had poor accessibility to township and village hospitals. The reasons for poor accessibility at city hospitals were long waiting time (34.7%), difficult registration (33.6%) and inconvenient transportation (31.8%). When it comes to healthcare expenses, we found 74.9% of the respondents believed that the healthcare fees were far too high, especially the drug prices (78.3%) and check-up fees (61.5%) (Figure 2).

### The public satisfaction level

Table 2 presented the enrollees’ satisfaction rate of the current NCMS policy and the effect of high healthcare service fees on their level of satisfaction. The majority (68.6%) of participants felt satisfied with the current NCMS policy. And a considerable proportion of enrollees’ compliant of high healthcare service fees, accounting for 73.4%. In addition, among the people believing that the NCMS was barely acceptable or even worse, 82.4% of them held that the healthcare service fees were fairly high.

**Table 2:** **The influence of high healthcare service fees on the NCMS**

High healthcare service fees	Satisfaction level with NCMS					
	Very satisfied	Satisfied	Barely acceptable	Dissatisfied	Very dissatisfied	Total
Yes	69(16.8%)	198(48%)	104(25.3%)	32(7.7%)	9(2.2%)	412(73.4%)
No	41(27.5%)	77(51.5%)	26(17.6%)	5(3.4%)	0(0%)	149(26.6%)
Total	110(19.6%)	275(49%)	130(23.2%)	37(6.6%)	9(1.6%)	561

X^2^ = 16.2; n = 10;15.99 < P < 18.31;the high medical service is associated with the effect of NCMS (P = 0.9).

### The improvements of the NCMS

**Figure 3:**
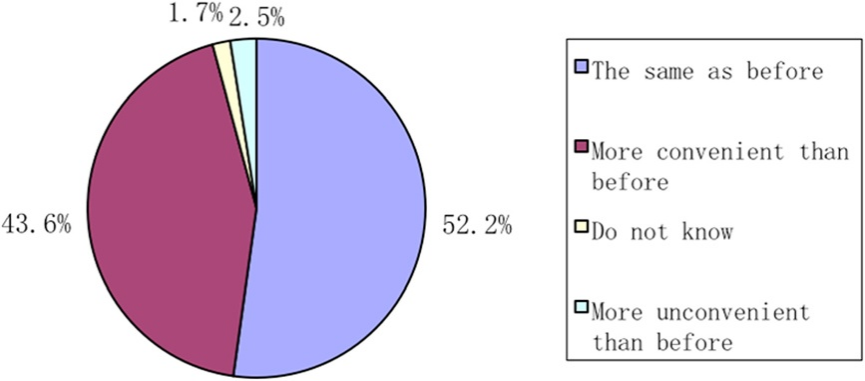
**The effect of the NCMS on inadequate healthcare services.**

**Figure 4:**
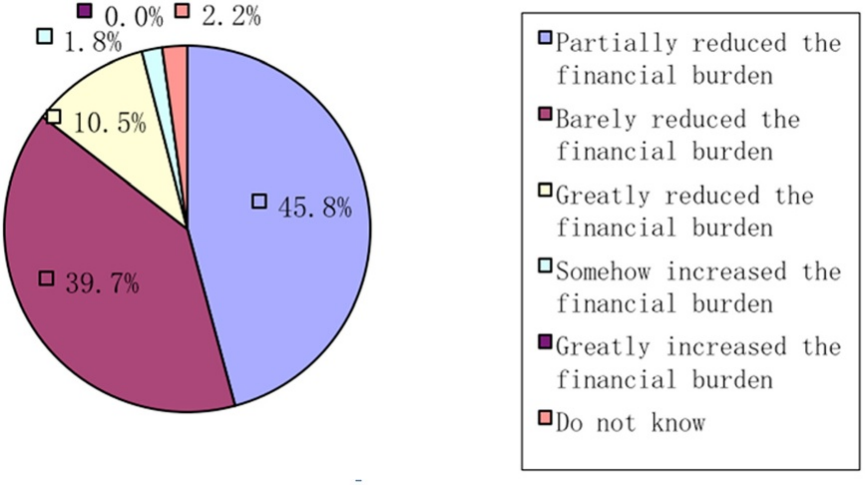
**The effect of the NCMS on high healthcare service fees.**

When asked about the improvements of the NCMS on the inadequate and overly expensive healthcare services, 43.6% of the respondents believed that it had been more convenient to receive healthcare services (Figure 3). In addition, 10.5% of the respondents thought that the NCMS had greatly relieved the financial burden of healthcare services (Figure 4), while 45.8% believed that there was still a wide gap between the final goal and the reality.

### Designated healthcare institution preferences

The majority (81.9%) of the respondents would prefer to go to the designated healthcare institutions, and 90.7% of the respondents lived nearby these institutions. When asked about where to seek a doctor when they got severe diseases, 72.3% of the respondents preferred the county hospitals, mainly for the reason of better skills and equipment; while they preferred village clinics (56.5%) and township hospitals (23.2%) when they got minor disease mainly for the reason of convenience; 15.4% of the respondents would rather buy some drugs and treat themselves than go to the hospitals (Table 3).

**Table 3:** **Participants’ views and utilization of healthcare institutions**

Variables		N	Percentages
Prefer to go to designated hospitals	Yes	462	81.9
	No	32	5.7
	Only for severe diseases	71	12.6
Satisfied with at least one healthcare institution	Yes	117	20.3
	No	460	79.7
Preference when get severe diseases	Municipal hospitals	108	18.0
	County hospitals	435	72.3
	Township hospitals	29	4.8
	Village clinics	14	2.3
	Others	16	2.6
Preference when get minor diseases	Municipal hospitals	0	0.0
	County hospitals	29	4.9
	Township hospitals	138	23.2
	Village clinics	337	56.5
	Others	92	15.4
The nearest healthcare institution	Designated health institution	495	90.7
	Non-designated health institution	51	9.3
Irrational medical treatment	Yes	208	42.9
	No	244	50.3
Frequency of minor diseases	No idea	33	6.8
	Ofen	159	21.7
	Occasionally	242	41.3
	Hardly	185	31.6
See a doctor when get minor diseases	Ofen	315	53.8
	Occasionally	89	15.2
	Hardly	182	31.3

Note: As some respondents did not fill in all the options, the total number of each category is not 602.

### Attitudes toward the designated healthcare institutions

In terms of service attitude, village clinics (63.3%) were thought to provide the best service, while county hospitals (21.8%) were voted to provide bad services. With regard to equipment, city and county hospitals were considered better equipped (65.3%) and they were with higher medical skills (58.3%). One interesting finding was that some village clinics seemed to provide some door-to-door services (26.2%), while the majority of designated healthcare institutions did not. In addition, a considerable number of the designated hospitals were considered to have higher drug prices than the average market prices (56.8% of county hospitals; 52.5% of township hospitals; 43% of village hospitals). Details were listed in Table 4.

**Table 4:** **Participants’ opinions on designated healthcare institutions**

Variables		County hospitals		Township hospitals		Village clinics	
		N	Percentages	N	Percentages	N	Percentages
Service attitude	Good	269	44.7	313	52.0	381	63.3
	Average	160	26.6	163	27.1	207	34.4
	Bad	131	21.8	45	7.5	25	4.2
Medical equipment	Good	393	65.3	150	24.9	68	11.3
	Average	128	21.3	287	47.7	190	31.6
	Bad	13	2.2	101	16.8	302	50.2
Skills	Good	351	58.3	115	19.1	95	15.8
	Average	117	19.4	310	51.5	165	27.4
	Bad	17	2.8	94	15.6	236	39.2
Door-to-door services	Yes	11	1.8	38	6.3	158	26.2
	No	518	86.0	564	93.7	444	73.8
Drug prices are higher than the market prices	Yes	342	56.8	316	52.5	259	43.0
	No	99	16.4	122	20.3	206	34.2

Note: As some respondents did not fill in all the options, the total number of each category is not 602.

## Discussion

### Principal findings

Our study provides new evidence on the effect of NCMS on the beneficiaries and their current state of satisfaction. In general, the NCMS policies have enjoyed high satisfaction rate, but many participants believed that there would be a long way in achieving its goal of preventing catastrophic poverty. The high healthcare service fees and inadequate healthcare services are still of great concern. The NCMS reimbursement package could not alleviate the financial pressure due to the high check-up fees and drug prices at the designated hospitals. Even though the village clinics were considered to provide the best healthcare services, people preferred higher level of designated hospitals when they got severe diseases, namely the county or even city hospitals. The long waiting hours and difficult registration process in these hospitals had been complaint constantly.

### The effect of the NCMS on healthcare service accessibility

According to our results, the majority of the NCMS enrollees would come to the designated hospitals for medical care when they get sick, and a considerable number of participants believe that seeking medical care has been more convenient. It is consistent with several previous studies [19, 20], reflecting the problem of inadequate healthcare services has been eased to certain extend. As found in our study, over 90% of the participants lived nearby the designated hospitals, indicating that the geographic accessibility has been improved much in the rural area.

Along with the great improvements, nearly one-third of the rural residents believed that the issue of inadequate healthcare services required further improvement, especially in county hospitals. It could be explained by the imbalanced distribution of healthcare resources. In terms of the city hospitals, where there are more advanced equipment and highly skilled providers, the large urban population drives up the overall demand for healthcare services. In that case, the limited city healthcare services may not meet the needs of the rural area. In addition, the weakened social role of township and village clinics will cause a lack of cost-effectiveness and the corresponding social-effectiveness [21], leading to low utilization of the available healthcare resources. Our finding that the rural residents preferred city or county hospitals when they got severe diseases, while they preferred the village clinics or buying drugs themselves when they got minor diseases, reflected the prominent inability of township and village hospitals.

### The effect of the NCMS on healthcare service affordability

A large proportion of rural residents believed that the healthcare services were expensive, which reflected the imbalance between healthcare costs and people’s affordability [22]. The longitudinal study from 2003 to 2005 by Chinese Ministry of Health and World Bank indicated that inpatient cost per case increased by 30% after people were insured [23]. In our study, nearly one-fifth of the respondents held that the high healthcare fees led to the ineffectiveness of the NCMS. The high healthcare fees were due to the high drug prices and high check-up expenses. Nearly 60% of the rural residents believed that the drug prices in designated hospitals were higher than the market prices, and the higher level of hospital, the higher the drug prices. In spite of the increasing reimbursement rate, the policy reimbursement rate for inpatient care is much higher than that in the outpatient care. Due to the higher price for inpatient care as well as the existence of reimbursement deductible, the poorer enrollees, especially these with chronic diseases, might not be well protected by the NCMS to receive proper medical care in time due to financial reasons. The poor were more likely to seek informal and less qualified providers, or resort to self-treatment when they were ill [24]. Other studies have also proved that unmet healthcare needs and service avoidance were more prevalent in the lowest socio-economic status households than in the highest socio-economic status households [25, 26]. Our finding provided a hard evidence for the pro-rich inequality for inpatient service utilization proposed by Yuan et al [27].

The NCMS has features like great publicity, quick financing and stable drug prices [28]. However, according to our study, the rural residents had a low level of satisfaction with designated hospitals. Only 20% of the rural residents felt satisfied with their designated hospitals, while nearly half of them believed that designated hospitals had certain irrational medical treatments like over-prescribing drugs. As the NCMS encourages people to seek healthcare services, providers may over-prescribe drugs and high-tech cares due to its fee-for-service payment system and the limited financing for health facilities from the government [29]. Furthermore, 40% of the participants had experienced irrational medical treatments like prescribing expensive drugs or repeated inspections [30]. According to Jiang et al., the overuse of injections, particularly the excessive unrestricted use of multiple injections, has become a big problem at the rural healthcare facilities in Sichuan [31]. The abuse of injections may partially be due to the misbelief that it would be more convenient and efficient than oral medications, but it is also linked with more revenues generated for healthcare facilities and medical stuff [32]. In other words, without proper public education or medical treatment restriction, the NCMS would play a limited role in reducing healthcare expenses.

### Policy implications

#### Redistribute healthcare resources

Rural China has 8.13 hospitals per million people, and 21.35 doctors and 1.75 beds per 1,000 people [33]. As was indicated in our study, the designated hospitals of the NCMS presented different healthcare service qualities and patients were prone to seek medical help form higher level of healthcare institutions like county hospitals instead of village clinics or township hospitals. Despite the government’s effort to restrain costs by encouraging patients to use township health centers, making the copayment amounts lower and patient reimbursement rates higher than at the county hospitals, the township hospitals failed to play its role of delivering rural health care and acting as a referral to the county hospitals. And the effort to promote the reasonable shunt of patients with a distinguishing subsidy turned out to be one of the causes of hard to receive healthcare service for the rural residents [34, 35].In this case, we suggest redistributing healthcare resources according to the coverage radius, population density as well as the general condition of every healthcare institution.

### Put more emphasis on medical equipment development

As high check-up fees play a dominant role in expansive healthcare fees, developing domestic medical equipment with low costs and properly allocating them to the rural areas would ease the problem. Furthermore, a standardized and networked electronic diagnosis and medical record system should be applied in different hospitals, which will avoid repeated tests, promote medical resource sharing and facilitate remote diagnosis. It could also benefit the patients who need transferring to higher levels of hospitals. The networked information system would also enable the administrative staff to trace the detailed information on healthcare service conditions in the rural areas.

### Establish a new talent flow mode

As indicated from our study, the township and villages hospitals were considered to provide better healthcare services but with lower clinical skills and more inadequate medical knowledge, which was one of the factors leading to irrational drug use. A study by Wang et al. showed that village doctors urgently needed more training on rational drug use [36], however, improving the skills of healthcare personnel was more critical to making full use of the NCMS [37]. To improve the medical skills of the working stuff at these hospitals, a long-lasting new talent flow mode to township and village clinics should be promoted. We suggest establishing a dynamic flow system consisting of medical professionals from city hospitals to county hospitals and from county hospitals to township and village clinics. The excellent medical stuff of every level of health institutions shall take turns working in healthcare institutions of other levels for a fixed period of time. To ensure the initiative of this policy, their performance shall be taken as an important criterion for professional qualification assessment, end-year bonus evaluation and title assessment. Simultaneously, the government shall provide more remedies to attract more medical staff of superior hospitals to rural medical institutions. With better skills, the village and township hospitals could attract more patients for their first visits and enhance their confidence in the hospitals. In addition, the village and township hospitals could exploit their advantages of low price and good service to the full to attract more patients.

### Limitations

The study has several limitations. Firstly, due to the limited sample size, certain bias could exist in some of our findings. And as the selection of the respondents focused on certain communities in the 21 villages, and the results may not be generalizable to the entire country, and could not fully represent the rural population. Secondly, some data in out study, like medical expanses, were collected on the basis of personal recall and could be prone to measurement errors. In addition, as we only focused our study in Liaoning province, and the NCMS policies could be somehow different from area to area, some of our findings might be limited to cities sharing similar policies.

## Conclusion

Our study suggests that the NCMS improved the situation of hard to receive healthcare services but did not reduce the high healthcare fees. The long waiting time during healthcare services remained to be the major problem in inadequate healthcare services. Furthermore, participants were unsatisfied with the NCMS designated hospitals, with the main reason of high expanses. The data in our study suggest that the designated hospitals of various levels presented different problems in aspects of service attitude, equipment, medical skills, service price, etc. The NCMS is clearly of great importance to reduce inaccessibility and unaffordability of healthcare services with the ultimate goal to reach universal health coverage. To achieve this goal, more studies shall be carried out in different areas to explore a more feasible policy that could be generalized on a broader scale and to get more hints for further policy making.

## Endnote

^a^Two-week prevalence rate = The number of patients in the first two weeks / The number of surveyed people 100%; the fourth National Health Services Survey showed the two-week prevalence rate in 2008 was 18.9%.
